# A large-scale proteogenomics study of apicomplexan pathogens—*Toxoplasma gondii* and *Neospora caninum*

**DOI:** 10.1002/pmic.201400553

**Published:** 2015-05-15

**Authors:** Ritesh Krishna, Dong Xia, Sanya Sanderson, Achchuthan Shanmugasundram, Sarah Vermont, Axel Bernal, Gianluca Daniel-Naguib, Fawaz Ghali, Brian P Brunk, David S Roos, Jonathan M Wastling, Andrew R Jones

**Affiliations:** 1 Institute of Integrative Biology, University of LiverpoolLiverpool, Merseyside, UK; 2 Institute of Infection and Global Health, University of LiverpoolLiverpool, Merseyside, UK; 3 Department of Biology, University of PennsylvaniaPhiladelphia, PA, USA

**Keywords:** Gene annotation, Microbiolgy, MS/MS, *N. Caninum*, Proteogenomics, *T. gondii*

## Abstract

Proteomics data can supplement genome annotation efforts, for example being used to confirm gene models or correct gene annotation errors. Here, we present a large-scale proteogenomics study of two important apicomplexan pathogens: *Toxoplasma gondii* and *Neospora caninum*. We queried proteomics data against a panel of official and alternate gene models generated directly from RNASeq data, using several newly generated and some previously published MS datasets for this meta-analysis. We identified a total of 201 996 and 39 953 peptide-spectrum matches for *T. gondii* and *N. caninum*, respectively, at a 1% peptide FDR threshold. This equated to the identification of 30 494 distinct peptide sequences and 2921 proteins (matches to official gene models) for *T. gondii*, and 8911 peptides/1273 proteins for *N. caninum* following stringent protein-level thresholding. We have also identified 289 and 140 loci for *T. gondii* and *N. caninum*, respectively, which mapped to RNA-Seq-derived gene models used in our analysis and apparently absent from the official annotation (release 10 from EuPathDB) of these species. We present several examples in our study where the RNA-Seq evidence can help in correction of the current gene model and can help in discovery of potential new genes. The findings of this study have been integrated into the EuPathDB. The data have been deposited to the ProteomeXchange with identifiers PXD000297and PXD000298.

## 1 Introduction

*Apicomplexa* are obligate intracellular parasites that are of great medical and veterinary importance. Key members in the phylum include *Plasmodium falciparum*, the causative agent of malaria; *Toxoplasma gondii*, responsible for severe congenital defects and causing fatalities in immunocompromised patients; *Cryptosporidium*, causing waterborne diarrheal disease in humans; *Babesia*, tick transmitted hemoprotozoan parasites that cause disease in cattle, horses, dogs, and humans; and finally, *Eimeria* and *Theileria* that account for severe diseases of food-producing animals in the United Kingdom and Europe. Apicomplexans are unified by an apical complex consisting of a cluster of apical secretory organelles such as rhoptries and micronemes, an apical polar ring and in some species a polarized microtubule organizing center called the conoid [1]. Host cell invasion is a key event for survival and replication of these parasites, which is conducted in a multistep process [2].

The genome information for these apicomplexan is maintained by the Eukaryotic Pathogen Database Resource Centre (EuPathDB: http://eupathdb.org) [3]. EuPathDB is an umbrella portal providing unified access to 11 specialized resources such as ToxoDB, PlasmoDB, etc. EuPathDB maintains genome sequences and annotations for these species along with a variety of pathogen genomics and functional genomics data and is an important resource for the apicomplexa research community. Among the first resources available at EuPathDB (formerly known as ApiDB back in 2005) was the genome of *Plasmodium falciparum*, which was the first apicomplexan genome to be sequenced [4]. This was followed by the sequencing efforts of many more apicomplexan species, including several other *Plasmodium* species [5–10], several strains of *T. gondii* [11], *Neospora caninum* [12], *Eimeria tenella* [10,11], *Babesia bovis* [13], *Cryptosporidium* [12,14,15], and *Theileria* species [16,17]. The efforts to sequence the *T. gondii* genome began in 2001 and ToxoDB release 2.0 had genome data for the ME49 and RH strains from a BAC clone-end sequencing project, an 8x random shotgun genome sequence, and expressed sequence tag (EST) assemblies [18]. The high-quality draft sequences and annotations for ME49, GT1, and VEG strains were completed by 2008 [11]. There are considerable manual curation efforts undertaken at EuPathDB and by the wider *Toxoplasma* community to produce high-quality gene models, as well as semi-automated reannotation based on experimental data. The high-quality genome of the closely related *Coccidian* parasite, *N. caninum* [12], has also been released recently, and the current *N. caninum* gene models were assembled using large-scale RNA-Seq datasets as a framework.

In this manuscript, we focus our attention on the current annotations of *T. gondii* and *N. caninum* only. We aim to study if proteomic evidence when used in conjunction with RNA-Seq evidence can help improve the current annotation of these species. The current gene model of *T. gondii* includes evidences from a number of previous MS-based studies performed to identify the proteome of *T. gondii*. These studies were typically based on the RH strain, which grows well and gives a high yield, and therefore is a well-accepted model for experimental studies. The first proteome studies either concentrated on studying a small number of tachyzoite proteins in detail by 2DE separation [19,20] or on studying subproteomes of rhoptry organelles [21], the apical complex [22], and the excreted-secreted proteome [23] of *T. gondii*. The multiplatform (2D electrophoresis, gel-LC linked MS/MS, and MudPIT) analysis of *T. gondii* tachyzoite proteome by Xia et al. [24] was the first global proteome analysis of *T. gondii*. The peptide evidence from this study was instrumental in the refinement of annotation of the genome including correct assignment of exon–intron boundaries [24]. A proteomic analysis of cytosolic and membrane fractions of *T. gondii* tachyzoites and the validation of gene models (TigrScan, TwinScan, Glimmer, and ToxoDB Release 4, NCBI nonredundant protein database) with EST and peptide evidence data by Dybas et al. showed 31–42% false negative rate for various gene models [25]. These were followed by two independent analyses of the proteome of oocysts and sporozoites of *T. gondii* [26,27]. All these data amount to a coverage of about 68% of predicted proteome of *T. gondii* [28]. The only proteome data publicly available to date for *N. caninum* include a gel electrophoresis based study identifying 26 differentially expressed proteins during tachyzoite to bradyzoite differentiation [29] and the analysis of the subproteome of rhoptry organelles [30,31]. The RNA-Seq evidences on the other hand have recently become available and have been included in the current gene models of *T. gondii* and *N. caninum* at ToxoDB. There has been availability of some published [32,33] and unpublished RNA-Seq datasets (http://toxodb.org/toxo/getDataset.do?display=detail) for both *T. gondii* and *N. caninum* that were used in the annotation.

Proteomics and RNA-Seq data provide complimentary evidence sets that can be exploited together for improvement in genome annotation. Next-generation sequencing techniques are already playing an increasingly important role in genome annotation. However, it is still difficult to unequivocally determine if a predicted gene actually produces a protein, or whether a predicted alternative splice of RNA is translated into protein. MS-derived proteomics data can play a direct role in genome annotation (proteogenomics), providing evidence that a given “official gene model” encodes a protein product, that a noncanonical (alternative) gene prediction is more likely to be correct, or that peptide evidence supports the discovery of new ORFs. Proteogenomics-based annotations utilize high-throughput MS/MS-based proteomic techniques to identify proteins present in a sample [34–36]. There are essentially four different ways of identifying peptide-spectrum matches from MS/MS: sequence database search [37,38], de novo sequencing [39,40], tag search [41,42], and spectral library search [43,44]. De novo sequencing does not require a sequence database to search against and thus could in theory be used to identify new peptides/proteins, but the error rates are generally too high for practical use in proteogenomics. The remaining three techniques are fundamentally dependent on the quality of the underlying database for identifying peptide sequences, with the sequence database search technique being the most common among them. The protein sequence databases are derived from gene annotations and a peptide sequence can only be identified by these methods if the corresponding gene sequence has been predicted correctly. The construction of a search database requires careful planning and one can simultaneously test a panel of gene models by combining evidences from independent computational and experimental sources. Gene models can differ from each other at a given locus in various ways, including prediction of start codon, presence–absence of coding regions, or precise intron–exon boundaries.

This manuscript takes a proteogenomics approach for studying the existing annotations of *T. gondii* and *N. caninum*, where we queried MS/MS datasets from eight different experiments using sequence databases comprising official gene models and high-quality RNA-Seq-assisted predictions. For this study, we reanalyzed a number of published datasets for *T. gondii* [24] and generated new high-throughput MS/MS datasets for both *T. gondii* and *N. caninum*. Apart from the protein identifications, we show that how this proteogenomics approach can provide a useful contribution in improving genome annotations of these species and can lead to discovery of novel genes. The “official” gene models for *T. gondii* and *N. caninum* were obtained from the latest releases available at EuPathDB and the RNA-Seq predictions were produced by the data available at the same repository. The proteomic identifications against the official gene models provide evidence for genes that result into a protein product, establish the validity of putative genes, and confirm predicted splicing events. Identifications against the RNA-Seq-based models on the other hand identify putative novel genes that have RNA-Seq evidence to support them but are missing from the official release, differ in the splicing of exons, or show alternate splicing from the canonical prediction. For both the species, we identified a large number of proteins and several targets for improvement in the current available annotation. The datasets and results have been made available at ProteomeXchange [45] and EuPathDB.

## 2 Materials and methods

### 2.1 Sample preparation and datasets

#### 2.1.1 *Toxoplasma gondii* datasets

Datasets of RH strain of *T. gondii* 1DE, 1DE of soluble and insoluble fractions (1DE SFIF), 2DE, and multidimensional protein identification technology (MudPIT) were created for a previously published *T. gondii* proteomics study [24]. Briefly, for 1DE, 1DE SFIF, and 2DE, *T. gondii* RH tachyzoites were separated either by 1D SDS-PAGE on a 12% v/v acrylamide gel or 2DE using pH 4–7 linear gradient and pH 3–10 nonlinear gradient strips. In total, 129 contiguous gel slices from the1DE experiment, 50 contiguous gel slices from the 1DE SFIF experiment, and 1217 gel spots from the 2DE experiments were collected and digested with trypsin. The peptide mixtures were then analyzed on an LC-MS/MS platform—an LTQ ion trap mass spectrometer (Thermo-Electron, Hemel Hempstead, UK) coupled online to a Dionex Ultimate 3000 (Dionex Company, Amsterdam, The Netherlands) HPLC system. For the *T. gondii* MudPIT experiment, five tris-soluble replicates and four tris-insoluble samples of *T. gondii* RH tachyzoite were each subjected to MudPIT analysis using a quaternary Agilent 1100 series HPLC coupled to an LTQ-ion trap mass spectrometer (Thermo, San Jose, CA, USA) with a nano-LC ESI source. Datasets of *T. gondii* “Orbitrap whole cell lysate” and “Orbitrap 1DE” were newly created for this study using previously published sample preparation and MS protocols [46]. These datasets were generated from RH parasites in order to be consistent with the previously published datasets. In the “Orbitrap whole cell lysate” experiment, *T. gondii* RH tachyzoite proteins were solubilized and tryptically digested. In the Orbitrap 1DE experiment, *T. gondii* RH tachyzoites were separated by 1D SDS-PAGE on a 12% v/v acrylamide gel, from which 16 gel bands were excised and digested with trypsin. The digests were then pooled into eight samples for LC-MS/MS analysis. Peptide mixtures from both experiments were analyzed by online nanoflow LC using the nanoACQUITY-nLC system (Waters MS technologies, Manchester, UK) coupled to an LTQ-Orbitrap Velos (ThermoFisher Scientific, Bremen, Germany) mass spectrometer equipped with the manufacturer's nanospray ion source.

#### 2.1.2 *Neospora caninum* datasets

Datasets of *N. caninum* Orbitrap 1DE and Orbitrap 1DE-2 were collected from *N. caninum* LIV strain tachyzoites using the similar protocol described in *T. gondii* datasets. Briefly, 8 and 14 gel slices were, respectively, excised from Orbitrap 1DE and Orbitrap 1DE-2 experiments. Samples were then tryptically digested and analyzed by online nanoflow LC using the nanoACQUITY-nLC system (Waters MS technologies) coupled to an LTQ-Orbitrap Velos (ThermoFisher Scientific) mass spectrometer equipped with the manufacturer's nanospray ion source.

It is important to note that all datasets were generated from single life cycle stages of the parasites, where not all proteins are expected to be expressed.

### 2.2 Proteogenomic analysis pipeline

The proteogenomics analysis of the above dataset was performed by an automated software pipeline, the ProteoAnnotator (http://www.proteoannotator.org/), developed in our group. ProteoAnnotator is an open-source, platform-independent, toolkit for proteogenomics, which is compliant with formats from the Proteomics Standards Initiative [47,48]. The pipeline starts with genomic coordinates for gene models (GFF3 (generic file)) format, and mass spectra in MGF (MASCOT Generic) format. ProteoAnnotator embeds open-source search engines OMSSA [49] and X!Tandem [50], which are accessed via the SearchGUI wrapper [51]. Postprocessing is performed to control for FDR at the peptide level, followed by a bespoke protein inference algorithm/scoring approach, which is able to score the strength of evidence for improvements to gene models at a given locus and assemble peptides into protein groups. For general identifications of protein groups—1% FDR is implemented. For (protein group) identifications that suggest improvements to the current gene models, 5% FDR threshold is applied—as discussed in the ProteoAnnotator publication [47].

### 2.3 MS/MS search parameters

#### 2.3.1 Search parameters

The search parameters for all the datasets were fixed modification of carbamidomethylation of cysteine, variable modification of acetylation of the protein n-terminus, pyro-glu from n-terminal E, pyro-glu from n-terminal Q, and oxidation of methionine. A double missed trypsin cleavage was allowed. The product tolerance was set as ±0.5 Da and the precursor tolerance as 5 ppm and ±0.5 Da for Orbitrap and LTQ, respectively.

#### 2.3.2 Gene models

The MS data for *T. gondii* and *N. caninum* were searched against the protein database assembled from two different sources: the official gene models and predicted gene models supported by RNA-Seq evidences. The official gene models (release 10 - January 30, 2014) were obtained from EuPathDB. The gene model for *T. gondii* was obtained from http://toxodb.org/common/downloads/release-10.0/TgondiiME49/gff/data/ and the *N. caninum* official model was obtained from http://toxodb.org/common/downloads/release-10.0/NcaninumLIV/gff/data/. At the time of submission of this manuscript, the latest official gene models are available as release 12 (September 10, 2014). However, releases 10 and 12 are identical in terms of protein counts and sequences. The RNA-Seq-assisted alternate gene model was generated using a version of CRAIG (default parameters) [52] that integrates RNA-Seq data available at http://toxodb.org/. The RNA-Seq data were encoded as features derived from the genomic mapping of RNA-Seq read libraries as performed by GSNAP version 2013-08-19 (default parameters) [53]. Following the same basic strategy as in [52], we obtained a set of gene predictions for each existing library. We sought completeness by forcing CRAIG to predict at least one gene model for each *biologically significant* junction. Junctions with conflicting evidence, that is junctions that overlap other junctions, are deemed biologically significant when their support is at least 20% of the highest support found in any overlapping junction. Junctions with no conflicts are biologically significant when they are supported by at least three reads mapping to the region. The RNA-Seq compliant version of CRAIG is unpublished at the time of preparing this manuscript, but the binaries for the current version are available at http://roos8core.bio.upenn.edu/∼abernal/craig/craig-2.1.tar.gz. The search database was prepared by concatenating these gene models to the official gene models. The database was further appended by decoy sequences with a target–decoy ratio of 1:1 to create a final search database for performing the MS/MS searches. We have provided these databases in the ProteomeXchange submissions.

#### 2.3.3 Functional analysis

Sequences from identified RNA-Seq-derived gene model with additional peptide evidence as well as overlapping official gene models were analyzed using InterProScan 5 [54]. Examples where additional domains and functional signatures identified in the RNA-Seq-derived gene model were further analyzed and annotated using Clustal Omega [55] and ToxoDB.

## 3 Results and discussion

Table 1 presents the composition of each gene model in terms of number of predicted gene sets and the total amino acid count. It also presents the total number of proteins identified by our analysis of these datasets for each set of gene models, summarized across all experiments. In order to count the total number of proteins, we selected only a single *representative protein* from grouped isoforms, where ambiguity was present in peptide to protein inference as per the ProteoGrouper algorithm in [47]. To our knowledge, the total number of official proteins identified for both *T. gondii* and *N. caninum* is the largest set of proteins with MS/MS evidence reported in any single study. Our analysis identifies ∼35% of the total protein coding genes and ∼50% of the total proteins with proteomic evidence reported for ME49 (http://toxodb.org/toxo/showRecord.do?name=OrganismRecordClasses.OrganismRecordClass&source_id=tgonME49&project_id=ToxoDB). There is no proteomics evidence available for *N. caninum*, so this is the first study where we report 1273 proteins with MS/MS evidence (http://toxodb.org/toxo/showRecord.do?name=OrganismRecordClasses.OrganismRecordClass&source_id=ncanLIV&project_id=ToxoDB). Our analysis also suggests that there are 289 and 140 potential loci for *T. gondii* and *N. caninum*, respectively, that have support from RNA-Seq and proteomics data but have incorrect gene models or are missing from the current annotation. When further stringent thresholding (5% FDR) is applied, there are 191 and 101 proteins identified from the RNA-Seq-derived gene models for *T. gondii* and *N. caninum*, respectively, as discussed in “Identifications From the RNA-Seq models.”

**Table 1:** The composition of gene models and the number of representative proteins identified in total across our full data collection

Species	Gene model	Total database entries	Total amino acid count	Representative proteins identified as group leadersa)	Alternate loci with *q*-value < 0.05
*T. gondii*	Official	8322	6 669 204	2921	0
	RNA-Seq	86 699	37 847 722	289	191
*N. caninum*	Official	7122	6 054 032	1273	0
	RNA-Seq	13 777	8 158 875	140	101

a) A “representative protein” can encompasses more than one record (protein) from the protein database, incorporating the set of proteins that share the same set or subset of peptide identifications to avoid double counting of proteins with no independent evidence.

The complete list of identified proteins, along with corresponding peptide hits and locations, is provided in the result files available at ProteomeXchange. Additionally, all results have been loaded into EuPathDB, aligned against the most recent genome release. An example region on chromosome VIII can be viewed at: http://toxodb.org/cgi-bin/gbrowse/toxodb/?start=5924028;stop=5974027;ref=TGME49_chrVIII;width=800;version=100;flip=0;grid=1;id=d6546393407f30e0e323ffd34a77b61a;l=peptide_ToxoDB_18db2e_http_http://www.proteoannotator.org_datasets_Toxo10_official_peptides.gff_1%1EGene—where datasets can be viewed alongside RNA-Seq and other large-scale datasets (On GBrowse—Click “Select Tracks,” “Supplementary Data Provided by Users”—All On). Alternatively, the results can be loaded to any desktop-based genome browser by loading the GFF3 file from http://www.proteoannotator.org/datasets/Toxo10_official_peptides.gff.

### 3.1 Identifications from the RNA-Seq models

The conflict where a PSM identified a peptide that belonged to both the official and RNA-Seq models was resolved by using the ProteoGrouper algorithm in ProteoAnnotator, in which identifications are assembled into protein groups. The protein groups are assigned two types of score (see [47]): (i) the overall score for the (protein ambiguity) group (*PAG Score*) and (ii) the strength of evidence supporting identified loci not from the official gene models (noncanonical gene model score). In the case of a tie between an official model and an identification from the alternative (RNA-Seq) models (same set of peptides), the protein group leader is by default assigned as the official model and the *noncanonical gene model score* = 0. The sum of peptide scores uniquely assigned to the group is used to generate the *PAG Score*. The protein group list is ordered by *PAG Score* (including targets and decoys), and a 1% FDR (protein group-level) threshold is applied. This basis was used to generate the figures in Table 1 for official gene models. For any groups where peptides have uniquely been assigned to an RNA-Seq-derived protein, the *noncanonical gene model score* is the sum of all peptide scores within the group assigned to models other than the official gene models. All “decoy” peptides are by default not assigned to an official gene model, and thus contribute to the *noncanonical gene model score* for the corresponding decoy protein group list. As such, all decoy peptides/proteins have *noncanonical gene model score* >0. The protein group list is separately ordered by *noncanonical gene model score* and a 5% FDR (*q*-value) threshold is applied (since this is conservatively calculated from all decoy peptides/proteins, this step ensured that the weak identifications from the alternate models are filtered out—e.g. those supported by only a single weak peptide identification matched to one RNA-Seq model), giving a list of significant alternate loci supporting evidence for improvement in the genome annotation. The *q*-value threshold at 5% resulted in identification of 191 and 101 proteins from the RNA-Seq-derived gene model for *T. gondii* and *N. caninum*, respectively. There are number of proteins identified with high *noncanonical gene model scores* that have not been assigned a chromosome, for example tgondii-rna.Reid_tachy.day3.gene1112 (5 noncanonical peptides), tgondii-rna.Reid_tachy.day4.gene797 (17 noncanonical peptides), tgondii-rna.Saeij_Jeroen_strains.COUGAR.gene1934 (15 noncanonical peptides), etc. These identifications are examples of those cases that are missing from the official annotation and are potential candidates for new genes.

Figures 3 present three different cases selected from the list of alternate identifications that suggest various improvements for the official gene model, including a different start codon, an extended gene model, and a different splicing site.

**Figure 1:**
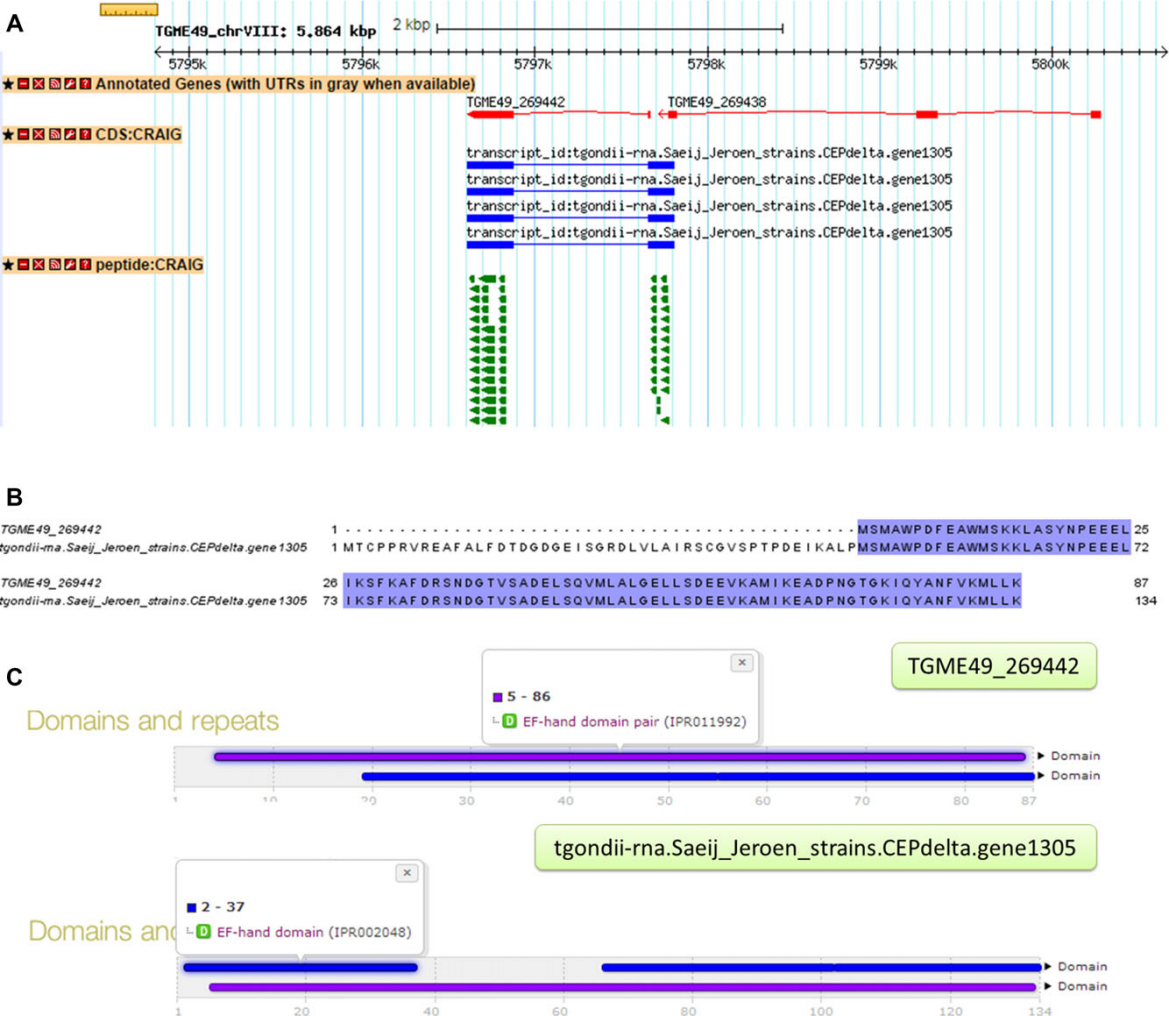
Peptide evidence indicating an alternate start codon. RNA-Seq-derived gene model tgondii-rna.Saeij_Jeroen_strains.CEPdelta.gene1305 was identified in our analysis, which has a different starting site to the official gene model TGME49_269442. (A) GBrowse screenshot of the alignment of the official gene model, RNA-Seq-derived gene model, and peptides identified. (B) Sequence alignment of official and RNA-Seq-derived gene model. (C) Comparison of InterProScan results between official and RNA-Seq-derived models where a complete EF-hand domain pair was identified in the RNA-Seq-derived model.

**Figure 2:**
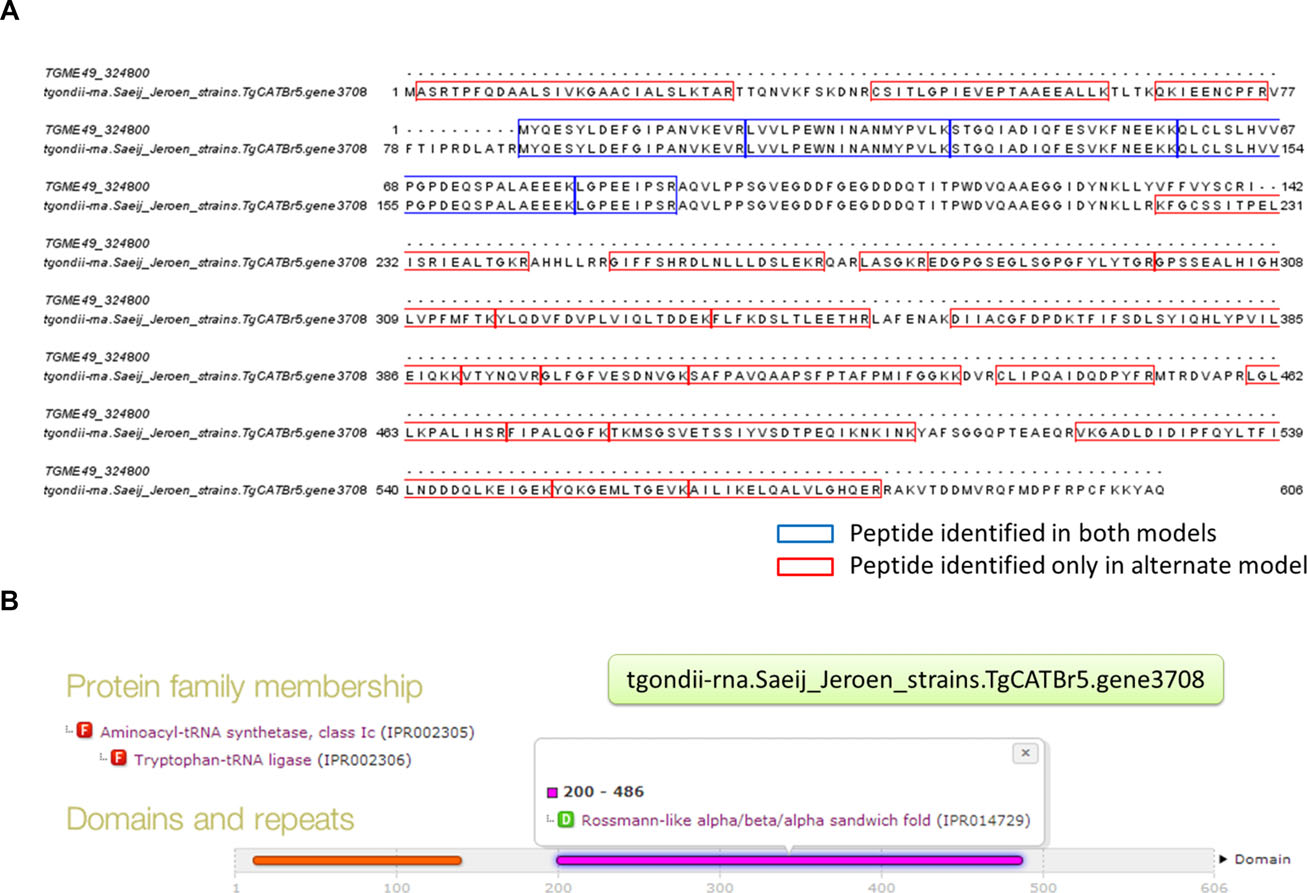
Peptide evidence indicating a suggested extension to the official gene model. RNA-Seq-derived gene model tgondii-rna.Saeij_Jeroen_strains.TgCATBr5.gene3708 was identified in our analysis that has a different starting site to the official gene model TGME49_324800. (A) Sequence alignment of official and RNA-Seq-derived gene model where peptides identified in both models are colored in blue and peptides identified only in RNA-Seq-derived model are colored in red. (B) Results from InterProScan indicate relevant domains detected in the RNA-Seq-derived gene model that are missing in the official gene model that has been annotated as tryptophanyl-tRNA synthetase.

**Figure 3:**
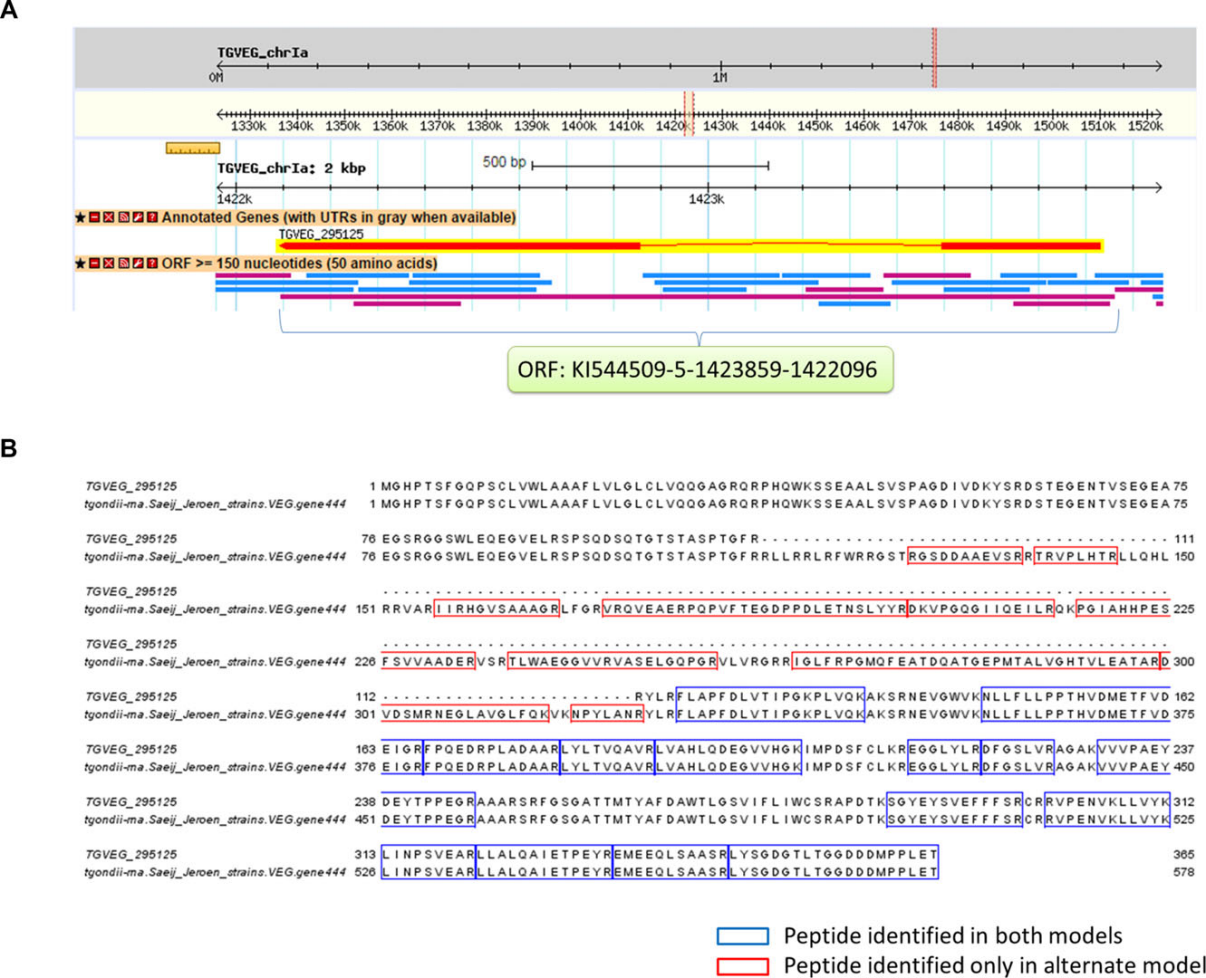
Peptide evidence indicating a different splicing site to the official gene model. RNA-Seq-derived gene model tgondii-rna.Saeij_Jeroen_strains.VEG.gene444 was identified in our analysis that has a different starting site to the official gene model TGVEG_295125. (A) GBrowse screenshot showing ORF KI544509-5-1423859-1422096 expands the intron region of the official gene model. (B) Sequence alignment of official and RNA-Seq-derived gene model where peptides identified in both models are colored in blue and peptides identified in the intron region of official gene model are colored in red.

Figure 1 presents a case of different start site picked by our analysis from two different datasets where two genes, TGME49_269442 and TGME49_269438, on chromosome VIII are in the immediate proximity of each other. The last exon of TGME49_269442 is within 116 base pairs of the first exon of TGME49_269438. The predicted gene model suggests a single transcript—tgondii-rna.Saeij_Jeroen_strains.CEPdelta.gene1305—that spans across the boundaries of both the genes. The presence of peptide evidence for this transcript suggests that these gene boundaries may be wrong. The official gene is annotated as putative calmodulin, a calcium-binding messenger protein. Calcium plays a critical role in several parasite-specific functions including host cell invasion and egress [56]. As shown in Fig. 1C, domain analysis suggested that, with a different starting site, the RNA-Seq-derived model possesses a complete EF-hand domain pair where the official model only includes a single EF-hand unit. Pairing of EF hands is thought to stabilize the protein and increase the affinity toward calcium [57], the RNA-Seq-derived model here may present a biologically more complete calmodulin protein with better calcium-binding efficiency.

A case of an extended gene model is shown in Fig. 2 where the official gene model for TGME49_324800 has no domain detected in domain analysis, but has been annotated as tryptophanyl-tRNA synthetase. The RNA-Seq-derived model has both Threonyl/alanyl tRNA synthetase, class II-like, putative editing domain (in red, position 12–139, IPR018163), and a Rossmann-like alpha/beta/alpha sandwich fold (IPR014729) detected. The official model lies between the end of the first domain and beginning of the second domain, which might be the reason for the current product name annotation. With an extended gene structure, the RNA-Seq-derived model has two full domains to support the product name and to make the annotation more complete.

Our analysis has also detected cases for different splice sites, an example for gene TGVEG_295125 is shown in Fig. 3. TGVEG_295125 is annotated as rhoptry protein ROP4 with two exons (see Fig. 3A). However, sequence alignment has shown that an ORF (ToxoDB: KI544509-5-1423859-1422096) can be found to expand the full length of the gene, including the intron region. In fact, 17 nonofficial peptides were identified from five datasets that mapped to the intron region of the official gene model (Fig. 3B), which suggested that RNA-Seq-derived model tgondii-rna.Saeij_Jeroen_strains.VEG.gene444 represents the actual protein expressed in this region. Interestingly, tgondii-rna.Saeij_Jeroen_strains.VEG.gene444 aligns perfectly with ROP4 gene sequence deposited in GenBank (version: AAU87405.1; GI: 52788873), which was independently identified by cross-reacting mAbs [58]. Together, our analysis provides evidence that TGVEG_295125 should be annotated as a single exon gene with no splice site in the middle.

The expression evidence of alternate gene structures provided by our analysis allowed us to identify new functional features in a gene. For example, a TRAM/LAG1/CLN8 homology domain was identified in RNA-Deq derived model tgondii-rna.Gregory_VEG_mRNA.hour16.gene5326, suggesting additional functions of the official gene TGME49_295080, which is annotated as a hypothetical protein. Signal peptides were also identified in RNA-Seq-derived models, which were missing in the equivalent official gene models, such as tgondii-rna.Saeij_Jeroen_strains.BOF.gene2384 versus TGME49_214220 and tgondii-rna.Gregory_VEG_mRNA.hour16.gene1072 versus TGME49_254470, both of which were annotated as hypothetical proteins. These additional features identified in our study would provide valuable information for functional annotation of the genome.

### 3.2 Description of available result files

The MS/MS datasets and result files are available at the ProteomeXchange Consortium with the dataset identifiers PXD000297 and PXD000298. There are six types of data files submitted at ProteomeXchange for both *T. gondii* and *N. caninum* datasets. These files are of types—raw MSMS files (.raw), peak list files (.mgf), search results in native OMSSA and X!Tandem formats (.omx and .xml), search results in mzIdentML format with various scores (.mzid), annotation files with integrated peptide evidences (.gff), and summary files (.csv). Each file name starts with a prefix representing the dataset it belongs to. The linkage is also available via ProteomeXchange interface. The datasets for *T. gondii* are denoted by self-explanatory prefixes—1DE, 2DE, MPIT, Orb-1DE, and Orb-WL. The datasets for *N. caninum* are prefixed by NORB-1 and NORB-2 for Orbitrap 1DE and Orbitrap 1DE-2 datasets, respectively. The GFF and CSV (comma separated values) files are of direct relevance to annotators. The GFF files exist in two forms, one for the peptides mapped on the official model, and another for the peptides mapped on the RNA-Seq model. The GFFs that correspond to the official model are named with a suffix “_gff_A,” whereas the GFFs corresponding to the RNA-Seq model are named with a suffix “gff_B,” in same manner as our search database was prepared. As an example, the GFFs produced by searching the 1DE dataset for *T. gondii* are named as 1DE_mapped_gff_A.gff (for official model) and 1DE_mapped_gff_B.gff (for RNA-Seq model). These GFFs can be directly loaded in a genome browser of choice, or can be uploaded at the GBrowse custom track interface of ToxoDB (http://toxodb.org/cgi-bin/gbrowse/toxodb/). There are five CSV files for each dataset and are prefixed in the same manner as above to identify the dataset they belong to. The type of information in the CSV files can be identified by the particular suffixes the files have in their names as described in [47]. A list of representative proteins from each experiment and the final counts are provided in the Supporting Information Files 1 and 2 for *T. gondii* and *N. caninum*, respectively. The identifications from the RNA-Seq-derived model can be loaded at the GBrowse interface of ToxoDB using Supporting Information File 3. The file contains the predicted transcripts and identified peptides. The InterProScan results for the RNA-Seq-derived identifications are listed in Supporting Information File 4. A genome-wide distribution plot of suggested correction sites for *T.gondii* can be seen in Supporting Information File 5.

## 4 Concluding remarks

MS-based proteomics has an important role to play in successful annotation of gene models. Our analysis of *T. gondii* and *N. caninum* not only identifies many known protein-coding genes but also provides a framework for validation of hypothetical and putative proteins. Our study provides evidence that further improvements to the existing gene annotation models for these species are possible, and helps in pin-pointing the locations in the genome, which may require a revision. The inclusion of RNA-Seq predictions in our proteogenomics approach enables us to harness the combined power of genomics and proteomics data for discovery of new genes that are not part of the current annotation. We anticipate that the inclusion of this evidence into the routine curation process will assist in the generation of accurate gene models released at the genome databases for these important species.

An important aspect of our approach is the reproducibility of the entire process facilitated by the availability of the ProteoAnnotator software toolkit. The tools and results developed for this study have been installed locally at the EuPathDB database and will shortly be used as part of a standard build pipeline for adding proteomics data to the various databases held within different subsites. The findings of our study for both *T. gondii* and *N. caninum* genomes are also integrated on the ToxoDB and are part of standard query interface. The full dataset and result files are available in public domain at ProteomeXchange.

## Abbreviations

1DE SFIF: 1DE of soluble and insoluble fractions
CSV: comma separated values
EST: expressed sequence tags
EuPathDB: Eukaryotic Pathogen Database
GFF: generic file format
MudPIT: multidimensional protein identification technology

## Supporting Information

As a service to our authors and readers, this journal provides supporting information supplied by the authors. Such materials are peer reviewed and may be re-organized for online delivery, but are not copy-edited or typeset. Technical support issues arising from supporting information (other than missing files) should be addressed to the authors.

Table S1 Protein count per dataset

Table S2 Protein count per dataset

Table S3

Table s4

Figure s1
