# Supplementary figures and images for: A large-scale proteogenomics study of apicomplexan pathogens—Toxoplasma gondii and Neospora caninum
Source: Proteomics. 2015 May 15;15(15):2618–28. doi: 10.1002/pmic.201400553 (PMC4692086; doi:10.1002/pmic.201400553)

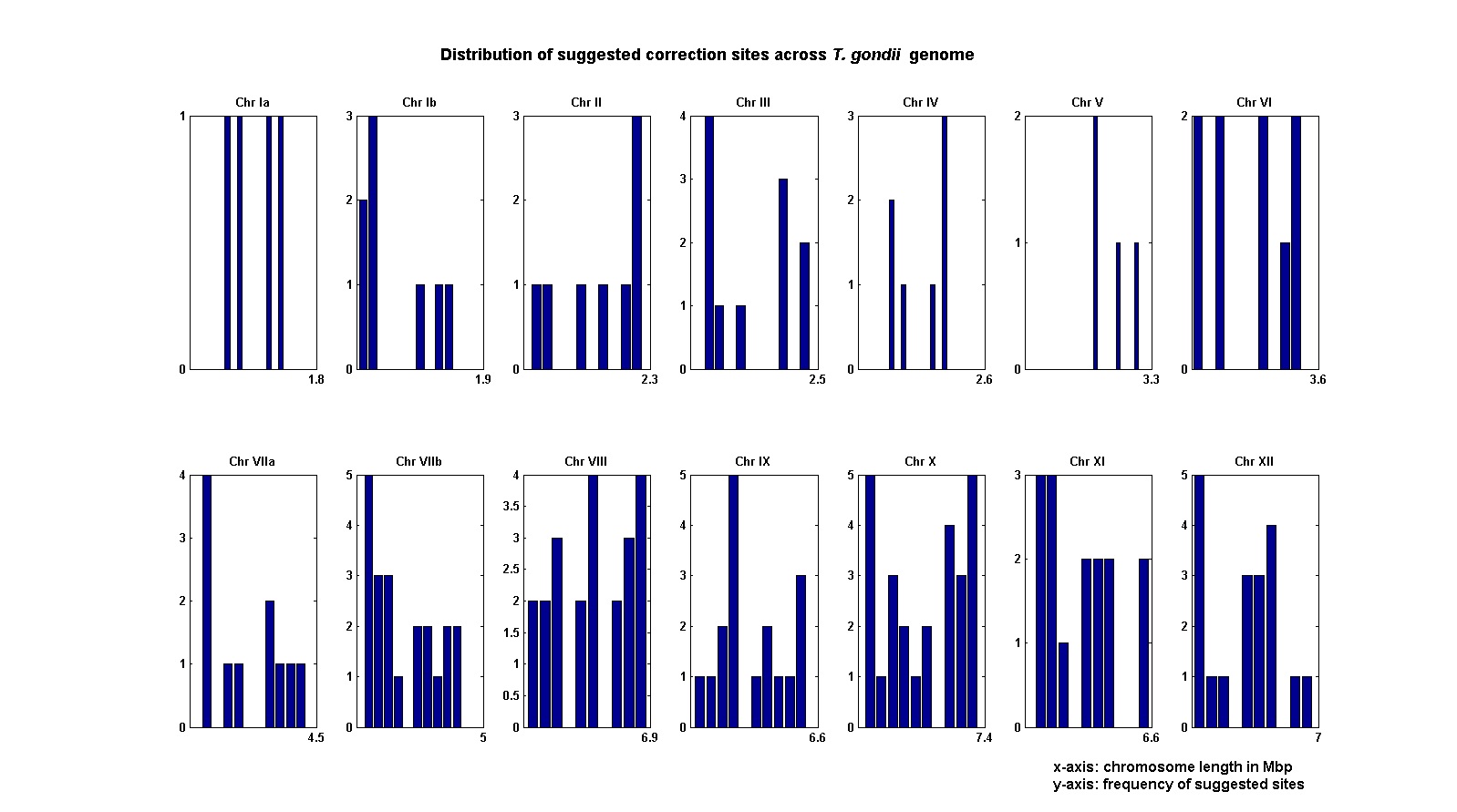

Supplement: Supplementary file 5 — Figure s1 [file pmic0015-2618-sd5.jpg]
